# The dynamics of male-male competition in *Cardiocondyla obscurior* ants

**DOI:** 10.1186/1472-6785-12-7

**Published:** 2012-06-15

**Authors:** Sylvia Cremer, Masaki Suefuji, Alexandra Schrempf, Jürgen Heinze

**Affiliations:** 1 Evolution, Behaviour & Genetics, Biology I, University of Regensburg, Regensburg D-93040, Germany; 2 Present address: Evolutionary Biology, IST Austria (Institute of Science and Technology Austria), Klosterneuburg A-3400, Austria

**Keywords:** Sexual selection, Male-male competition, Fighting, Cuticular hydrocarbons, Detection abilities, *Cardiocondyla* ants

## Abstract

**Background:**

The outcome of male-male competition can be predicted from the relative fighting qualities of the opponents, which often depend on their age. In insects, freshly emerged and still sexually inactive males are morphologically indistinct from older, sexually active males. These young inactive males may thus be easy targets for older males if they cannot conceal themselves from their attacks. The ant *Cardiocondyla obscurior* is characterised by lethal fighting between wingless (“ergatoid”) males. Here, we analyse for how long young males are defenceless after eclosion, and how early adult males can detect the presence of rival males.

**Results:**

We found that old ergatoid males consistently won fights against ergatoid males younger than two days. Old males did not differentiate between different types of unpigmented pupae several days before emergence, but had more frequent contact to ready-to-eclose pupae of female sexuals and winged males than of workers and ergatoid males. In rare cases, old ergatoid males displayed alleviated biting of pigmented ergatoid male pupae shortly before adult eclosion, as well as copulation attempts to dark pupae of female sexuals and winged males. Ergatoid male behaviour may be promoted by a closer similarity of the chemical profile of ready-to-eclose pupae to the profile of adults than that of young pupae several days prior to emergence.

**Conclusion:**

Young ergatoid males of *C. obscurior* would benefit greatly by hiding their identity from older, resident males, as they are highly vulnerable during the first two days of their adult lives. In contrast to the winged males of the same species, which are able to prevent ergatoid male attacks by chemical female mimicry, young ergatoids do not seem to be able to produce a protective chemical profile. Conflicts in male-male competition between ergatoid males of different age thus seem to be resolved in favour of the older males. This might represent selection at the colony level rather than the individual level.

## Background

Male age is an important predictor for engagement and success in male fighting [1]. Middle-aged males often win in male-male competition, since males gain experience with age, but also loose strength when getting older. Sexually non-active males are typically not attacked, as they have not yet developed morphological secondary sexual characters, do not display the behavioural repertoire of mature males, or do not enter mating areas [1].

Male-male competition is extremely pronounced in the ant genus *Cardiocondyla*. Whereas mating in many ants occurs during a mating flight [2], sexuals of *Cardiocondyla* mate in their natal nests [3,4]. Intra-nest mating leads to a “seraglio situation” [5] like in several species of fig wasps and parasitoid wasps [6,7], *i.e.*, it allows males to monopolise matings with newly emerging female sexuals. This selects for rigorous fights even among closely related males [8-10].

*Cardiocondyla obscurior* (Wheeler, 1929) shows a conspicuous male diphenism with winged and wingless males. The wingless (“ergatoid”) males mate locally within the maternal colony and are well adapted for fighting in morphology and behaviour [4,11-15]. They patrol through the nest, grab other ergatoid males with their elongated, sickle-shaped mandibles, and mark them chemically with hindgut secretions [16]. The besmeared males are killed by workers within minutes to hours, so that only a single adult ergatoid male is present per colony, even though many more may be reared [17]. Winged males, in contrast, resemble the typical ant male in morphology and behaviour: they are docile and a few days after adult emergence disperse from their natal nests [4,11-14]. Before dispersal, they may also mate with female sexuals inside the nest. Though quite vulnerable, they are normally not attacked by their wingless rivals but appear to be protected through chemical female mimicry [18,19].

The different parties in male-male competition obviously have conflicting interests concerning their detectability. Dishonest female mimicry by winged males might be stable in evolution because winged males are only sporadically produced under environmental stress [11,12]. Winged males are thus rare compared to the constantly reared female sexuals, and it might be more costly for a wingless male to mistakenly kill a female sexual than sparing an occasional winged competitor [20]. Ergatoid males would presumably benefit from identifying other ergatoid males when these are still relatively defenseless and killing them is without risk. In contrast, young ergatoid males would increase their chance of surviving the critical first few days of their adult life by hiding their identity.

We therefore investigated how long young males are vulnerable and how early adult males can detect the presence of emerging rivals in their nests, *i.e.*, whether detection is already possible in the pupal stage before eclosion. Behavioural observations were supplemented by the analysis of surface chemicals of young males, female sexuals, and workers, and the respective pupal stages.

## Methods

### Study organism

*Cardiocondyla obscurior* is a species that is neither endangered nor protected. Ants were collected from a single unicolonial population [13] in Una, Bahia, Brazil as allowed by Brazilian authorities (permit RMX 004/02) and reared in the laboratory as described in [11]. All experiments comply with the laws of Germany and Europe. Research with ants does not require approval by an ethics committee.

### Male fighting

We observed 24 fights between pairs of ergatoid males. Males were removed from their original nests and placed in a small colony containing 10–20 workers. It was previously found that the order in which males were introduced to the workers does not affect the outcome of fights (SC unpublished observation). In the encounters, one male was older than three days (“old male”), whereas the age of the younger male varied between immediately after emergence (d0, *n* = 10), one-day old (d1, *n* = 7), and two-day old (d2, *n* = 7). For each fight we determined, which male first attacked the other, how often both males bit and besmeared one another, how often the workers bit either male, which male died or was the winner of the competition, as well as the total duration of the fights. We analysed overall 3x2 contingency tables by Fisher’s exact test (VassarStats). Posthoc tests were performed as Fisher’s exact tests on all three pairwise 2x2 comparisons, using a Bonferroni-adjusted significance level α = 0.017. Behavioural frequencies and fight durations were analysed by non-parametric Kruskal-Wallis tests followed by all-pairwise posthoc Dunn’s tests (SigmaStat 2.03).

### Interaction of adult males with pupae

We observed the behaviour of single ergatoid males towards pupae in an arena (diameter 4 cm, height 0.5 cm) by scan sampling (each scan taking one to several seconds [21]) every 30 min over a period of five hours (*n* = 10 observations per male). We placed one worker pupa, one female sexual pupa, one winged and one ergatoid male pupa onto fresh filter paper in random order in different quadrants of the arena. Pupae were either unpigmented and thus a few days prior to emergence (“white pupae,” *n* = 12 replicates), or darkly pigmented and thus near adult eclosion (“dark pupae,” *n* = 12). Before the experiment, we determined with empty arenas (control, *n* = 7) that the ergatoid males did not have any *a priori* bias for any of the quadrants. We observed for each replicate, how often the ergatoid male was in direct contact with the different pupae and how frequently it performed antennation behaviour, biting, smearing, and copulation attempts. Contact frequencies were arcsine square root transformed and subjected to ANOVA followed by all-pairwise Protected Fisher’s LSD posthoc comparisons (SPSS Version 20). The occurrence of biting, smearing and copulation behaviour in interactions of the ergatoid males to the dark pupae was tested for differences in brood type by Fisher’s exact test based on 4x2 contingency tables (VassarStats). Figures are based on raw data.

### Chemical analysis

We determined the cuticular hydrocarbon profiles of white and dark pupae and one-day old ants of both female castes (workers, female sexuals) and both types of males (winged, ergatoid; *n* = 5 replicates each) by gas chromatography (GC; Agilent 6890 N GC with flame ionization detector equipped with a HP-5 column: 30 m * 0.25 mm * 0.25 μm). Cuticular compounds were identified based on their retention indices and previous mass spectrometry [19]. We extracted each sample (pupae: pools of five individuals; adults: single individuals) in 10 μl pentane (5 min in 1.8 ml vials with 200 μl glass inserts) and injected 5 μl of the extract into the GC. Initial oven temperature was 100°C, which was increased after 2 min to 180°C at 30°C/min, and then to 210°C at 10°C/min, and finally to 300°C at 4°C/min, and kept for 10 min (total run time: 40.17 min). Peak integration was performed with the program Chem Station. Pupae overlapped with the adult profile [19] consistently, *i.e.*, in all individuals, in only 11 peaks (see results). These were used for statistical data analysis after transformation of the standardized peak areas following [22] as Z_ij_ = log[X_i,j_/g(X_j_)], where X_i,j_ = standardized peak area i for the sample j and g(X_j_) = geometric mean of all peaks of the sample j. Transformed data were then subjected to a principal components analysis to reduce the number of factors. To determine how well the different groups were differentiated we performed a discriminant analysis with the principal components of eigenvalues > 0.8. Squared Mahalanobis distances were subjected to sequential Bonferroni correction due to multiple comparisons between all groups in the matrix. Multivariate statistical analyses were done with Statistica 6.0, permutation tests with the software PAST version 1.75b [23].

## Results

### Male fighting

**Figure 1:**
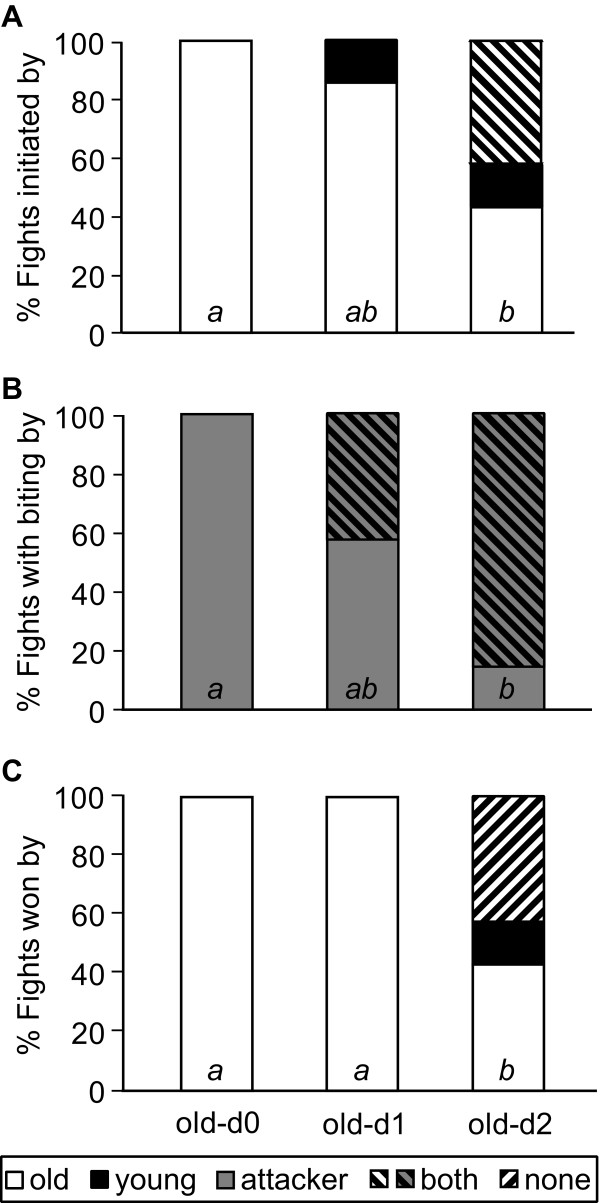
**Fight characteristics depending on the age of ergatoid males of the ant *Cardiocondyla obscurior*. A)** Fights were initiated more frequently by the older male when their opponent had eclosed on the day of fighting (old-d0) or was one day old (old-d1) than when the young male was two days old (old-d2). **B)** Biting was performed only by the attacking male when the opponent was less than one day old (old-d0), whereas both males attacked when the young male was older (old-d1 and old-d2). **C**) Fights were won exclusively by the older male when the rival was younger than two days (old-d0 and old-d1), yet in interactions between an old and a two-day old male, both males had similar chances of winning. Some fights resulted in the death of both males. Small italic letters indicate significance groups of the white/grey bars.

Fights lasted from ten minutes to 60 hours, with the duration not being significantly dependent on whether the old ergatoid males was paired with a freshly emerged (d0; median fight length, 25%-75% quartile range: 367 min, 89–475 min), a one-day old (d1; 420 min, 246–1104 min) or a two-day old (d2; 473 min, 349–1601 min) opponent (Kruskal-Wallis test *H* = 1.54, 2df, *P* = 0.46). The characteristics and outcome of the fights, however, strongly depended on the age of the two males, with the old male generally having an advantage over rivals one-day old or younger. Old males initiated the fights more frequently when their opponent was freshly emerged (100%, 10/10 fights) or one-day old (86%, 6/7 fights) than when the young male was already two days old (43%, 3/7 fights; Fisher’s exact test, *P* = 0.013; posthoc comparison d0-d2: *P* < 0.017, d0-d1 and d1-d2 n.s.; Figure 1A). Only in the latter case, both males occasionally attacked one another at the same time (43%, 3/7). Biting was never performed by freshly emerged males. In contrast, when an old male was paired with a one- or two-day old opponent, both the attacking and the attacked male were biting in 43% (3/7) and 86% (6/7) of the fights (Fisher’s exact test, *P* < 0.001; posthoc comparison d0-d2: *P* < 0.017, d0-d1 and d1-d2 n.s.; Figure 1B). Old males always won the fight against freshly emerged and one-day old males (10/10 and 7/7, respectively) but survived in only 43% (3/7) of fights against a two-day old rival (Fisher’s exact test, *P* < 0.007; posthoc comparison d0-d2: *P* < 0.017, d0-d1 and d1-d2 n.s.; Figure 1C). Interestingly, 43% (3/7) of these latter fights led to the death of both opponents, whereas the younger male was only able to win in a single of the observed fights (14%).

**Figure 2:**
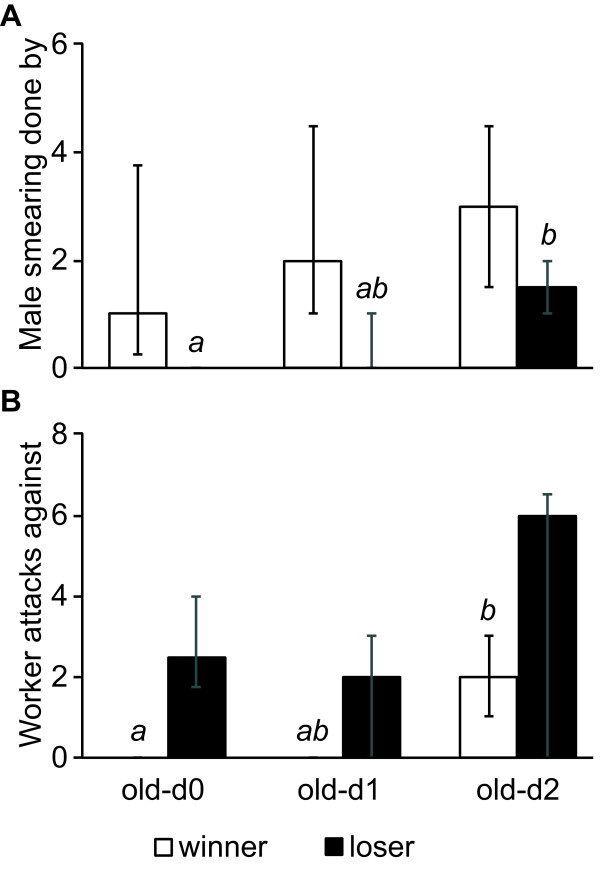
**Male smearing and worker attacks in male-male fights of the ant *Cardiocondyla obscurior*. A)** The frequency of observed male smearing performed by the later winner of the fight was independent of the age of the two males. Besmearing activity by the later loser increased with the age of the young male. **B)** Worker attacks towards the later loser of the fight did not vary with the age of the two opponents. The occurrence of worker biting against the later winner of the fight increased with the age of the young male. Bars represent median values and whiskers the 25% and 75% quartiles. Small italic letters indicate significance groups for significant overall differences (black bars in A and white bars in B).

In addition to biting, males besmeared their rivals with secretions from the anus, which elicited worker aggression. The frequency with which the later winner of the fight besmeared its rival was independent of male age (*H* = 1.89, 2df, *P* = 0.4; Figure 2A). In contrast, the prospective loser besmeared its opponent more frequently in fights between old and two-day old males than in fights with a freshly eclosed male. In the latter case, besmearing did never occur (*H* = 12.0, 2df, *P* = 0.002; Dunn’s posthoc test d0-d2: *P* < 0.05; d0-d1 and d1-d2 n.s.; Figure 2A). Worker attacks against the later loser did not differ between the three groups (*H* = 1.85, 2df, *P* = 0.49; Figure 2B), but were performed at higher frequencies against the later winner in encounters of an old with a two-day old male than with a freshly emerged male (*H* = 11.52, 2df, *P* = 0.003; Dunn’s posthoc test d0-d2: *P* < 0.05; d0-d1 and d1-d2 n.s.; Figure 2B). Fights between old and one-day old males showed intermediate frequencies of besmearing performed by the later loser and worker attacks towards the later winner.

### Interaction of adult males with pupae

**Figure 3:**
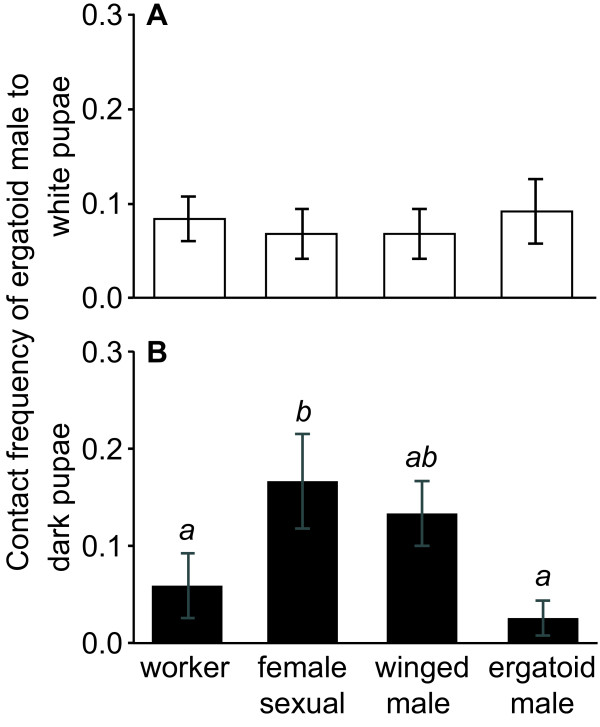
**Contact of adult ergatoid males towards pupae of all colony members in the ant *Cardiocondyla obscurior*. A)** Ergatoid males did not preferentially contact any type of pupae when all pupae where white and thus several days prior to emergence. **B)** Dark, ready-to-eclose pupae of workers and ergatoid males were contacted less frequently than female sexual pupae, as well as winged male pupae. Bars represent mean values and whiskers the standard error of the mean. Small italic letters indicate significance groups for the dark pupae that showed significant overall differences.

**Figure 4:**
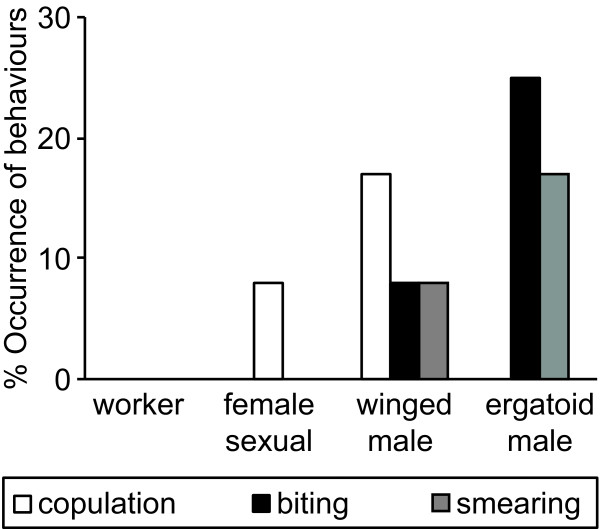
**Mating and fighting behaviour of ergatoid males towards dark pupae in the ant *Cardiocondyla obscurior.*** Ergatoid males performed copulation attempts towards dark female sexual and winged male pupae, which were close to emergence. Sporadic biting and smearing was observed against ergatoid male pupae and also against winged male pupae.

Ergatoid males showed similar contact rates to all young (white) pupae of the different types (worker, female sexual, winged and ergatoid male; ANOVA *F*_3,44_ = 0.22_,_*P* = 0.88; Figure 3A). Yet, when pupae were close to emergence (dark pupae), female sexual and winged male pupae were contacted at higher frequencies than workers and ergatoid males (ANOVA *F*_3,44_ = 3.94_,_*P* = 0.01; all pairwise posthoc comparisons by Protected Fisher’s LSD, *P* < 0.05 for comparisons worker-female sexual, female sexual-ergatoid male, winged male-ergatoid male; Figure 3B). Moreover, ergatoid males in rare cases performed behaviours to dark pupae shortly before emergence, which are expressed regularly against the respective adults, *i.e.*, copulation behaviour towards both female sexuals and winged males [4,14,18,24], and biting and smearing towards ergatoid males ([4,14]; Figure 4). Due to their low frequency of occurrence, these events did not significantly differ between the different types of pupae (Fisher’s exact test; copulation: *P* = 0.60; biting: *P* = 0.17; smearing: *P* = 0.60). Copulation behaviour was observed towards one female sexual pupa (8%, 1/12 replicates) and two winged male pupae (17%, 2/12). Biting was expressed towards 25% of ergatoid male pupae (3/12) and smearing towards 17% (2/12). Moreover, biting and smearing were observed towards one still white ergatoid male pupa. In addition, one dark winged male pupae was bitten and another one was besmeared by the ergatoid male (total 17%, 2/12, dark winged male pupae being attacked). In contrast to fights between adult ergatoid males, however, biting of pupae did not result in a permanent grab. Instead, the pupae were immediately dropped.

### Chemical analysis

**Figure 5:**
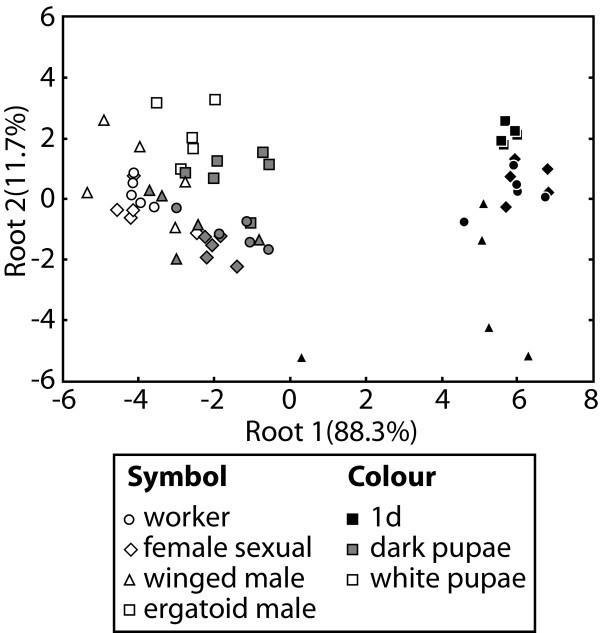
**Chemical analysis of cuticular hydrocarbon profiles of pupae and adults of the ant *Cardiocondyla obscurior*.** One-day old adults of *C. obscurior *ants were clearly separated by discriminant analysis from the pupae. Ready-to-eclose dark pupae showed a more similar chemical profile to adults than still unpigmented white pupae several days before eclosion.

Gas chromatography of cuticular hydrocarbons revealed that pupae and adults consistently shared only eleven peaks (C_25_; 11-meC_25_; 3-meC_25_; C_27_; 13-, 11-, 9-meC_27_; C_29:1_; C_29_; 15-, 13-, 11-meC_29_; 7-meC_29_; C_31:1_; 15-, 13-, 11-meC_31_). Principal components analysis of transformed and standardized peak areas resulted in two principal components with eigenvalues larger than 0.8, which together accounted for 90.6% of the variance. Discriminant analysis with these two principal components indicated a significant separation of the 12 different groups (white pupae, dark pupae, 1d old adults each of workers, female sexuals, winged males, and ergatoid males; Wilks' λ = 0.0127, *F*_22,94_ = 33.616, *P* < 0.0001). The cuticular profiles of pupae and adults were clearly separate (Figure 5), and the squared Mahalanobis distances between white pupae and the group centroids of the corresponding adults were larger than those between dark pupae and adults (permutation t-tests; workers: *P* = 0.003, female sexuals: *P* < 0.002, winged males, *P* = 0.064, ergatoid males: *P* = 0.053). Most misclassifications occurred between different types of pupae or between different types of adults and only a single adult was incorrectly classified as a pupa (a one-day old winged male as a dark female sexual pupa). Over all life stages, correct classifications occurred in 60% of the cases. Most female sexuals and ergatoid males were correctly classified, which matches the behavioural observations. Misclassifications occurred at similar frequencies among white pupae (9/20), dark pupae (8/20) and adults (7/20). Most squared Mahalanobis distances between ant types of the same age group were no longer significant after sequential Bonferroni correction. Exceptions involved adult winged males, which were separate from all other adults, dark ergatoid male pupae, which were different from dark female sexual pupae, and white ergatoid male pupae, which differed from white pupae of female sexuals and workers.

## Discussion

Our study shows that ergatoid males benefit from attacking emerging ergatoid rivals as soon as possible after eclosion, even if the latter do not yet engage in sexual activities. Older males consistently won fights against males of an age of one day or less, whereas they only survived 43% of the fights against two-day old opponents (Figure 1). Very young males hardly showed any fighting behaviour themselves (Figure 2). Their cuticula is not yet fully sclerotized and does not provide any protection against bites by older individuals (SC and AS, unpublished observation). Furthermore, the softness of their mandibles does not allow freshly emerged males to attack others. In contrast, two-day old ergatoid males were similarly active as older males and equally likely to initiate or win a fight. Encounters among older males led to pronounced fighting, in which occasionally both males were killed. By attacking freshly emerged ergatoid males, adult ergatoids therefore remove future competitors at the lowest possible risk. Old males that are unable to quickly detect an emerging rival male have a > 50% chance of being killed and replaced by a younger male.

Earlier eclosing males appear to have a competitive advantage over those that eclose later also in other species with fatal fighting and local mate competition (LMC), *e.g.*, parasitoid wasps [6,25]. In addition, differences in body size and previous fighting experience may affect the outcome of fights in parasitoids [25] and several insects without LMC [26-29]. However, these two factors appear to be much less important in fights between ergatoid males of *C. obscurior* than the timing of emergence (AS and SC, unpublished data).

Obviously, the advantage of early emergence results in a conflict of interest over the timing of the detectability of ergatoid males. On the one hand, it pays for an adult ergatoid to recognize and remove competitors as early as possible, as eliminating pupae is presumably even safer than killing freshly emerged ergatoids. On the other hand, young ergatoid males would benefit by concealing their identity until their cuticula is sclerotized and they have a reasonable chance of surviving a confrontation with an older male. Ergatoid males almost incessantly patrol through the nest and examine the brood pile for freshly eclosed ergatoids and young female sexuals [30,31]. Our behavioural analysis revealed that ergatoid males did not differentiate between the different types of unpigmented pupae several days prior to emergence, but showed increased contact to close-to emergence (dark) female sexual and winged male pupae (Figure 3). Also, whereas ergatoid males only showed antennation (*i.e.* detection [32]) behaviour towards young, unpigmented pupae several days before emergence, they were occasionally seen attempting to mate with female sexual and winged male pupae and attacking ergatoid male and also winged male pupae (Figure 4). Attacks against pupae were weak compared to encounters between adults and more intense behavioural observations will be required to determine how frequently these interactions occur and whether workers may kill besmeared ergatoid male pupae. Nevertheless, it appears that the conflict of interest has been decided in favour of the adult ergatoids.

The observed differences in ergatoid male behaviour towards unpigmented early pupae and dark pupae shortly before emergence may be based on their chemical signatures. We found that the cuticular hydrocarbon profiles of dark, ready-to-eclose pupae were more similar to those of adults than the profiles of white pupae (Figure 5). The chemical profile of dark pupae might therefore allow ergatoid males to determine the destiny of the emerging ant with at least some accuracy. Still, differences in the chemical profiles between ant types are likely not large enough to allow faultless determination. The costs of misidentifying and inadvertently killing a female sexual or worker pupa might explain why ergatoid males of *C. obscurior* did not kill ergatoid male pupae. This may be additionally pronounced by the fact that the chemical signal of pupae was also quantitatively less intense than that of adults: for GC analyses, we had to pool five pupae to obtain similar quantities of cuticular hydrocarbons as from single adults.

It has been shown previously that winged males of *C. obscurior* perform a chemical female mimicry that protects them against attacks of ergatoid males in the early days after emergence before they leave the colony [18]. Moreover, they adjust the timing of dispersal from the nest both to the availability of mating partners in the nest and the presence of ergatoid fighter males [33]. This might suggest that winged males may become a target for ergatoid male aggression once their chemical similarity to female sexuals fades [33]. In the present study, we found some indication that ergatoid males can already be lured to attempt copulation with dark winged male pupae prior to emergence. Whereas adult *C. obscurior* ants are characterised by a large set of 44 cuticular hydrocarbons [19], only 11 peaks overlapped between the pupal and adult profiles and were thus analysed in the current study. A comparison between the one-day old adults of the current study revealed that these shared 11 peaks are not identical with those peaks thought to be responsible for the chemical female mimicry [18,19].

## Conclusion

Though young ergatoid males would obviously benefit from concealing their identity they fail to do so. Whereas winged males perform successful female mimicry [18], ergatoid males may be morphologically too different from female sexuals (in size and the absence of wings) to allow successful female mimicry. The observed absence of self-concealment by young ergatoid males may be selected at the colony level rather than the individual level. In *C. obscurior,* female sexuals and ergatoid males are produced throughout the year and mating occurs in the natal nest. The colony as a whole might benefit from producing replacement ergatoids ready to mate with the continuously emerging young female sexuals in case the old ergatoid male dies. The cost of uninseminated female sexuals lies in a reduced colony growth due to a very low lifetime reproductive output of virgins. Even if uninseminated females can contribute to colony reproduction by laying unfertilised, male destined eggs, both their egg laying rate and lifespan are much below that of mated queens [34], whereas the latter even increase their egg laying efficiency with age [35]. In comparison to the loss of future colony growth through insufficient mating opportunities for female sexuals the costs of producing surplus ergatoid males might be relatively small. Furthermore, investment in surplus ergatoids is not completely lost as killed ergatoids are quickly ‘recycled’, *i.e.* dismembered, and fed to the larvae [36].

Despite their distinct chemical profile, ergatoid males may occasionally remain undetected because a) they are quite inactive during the first hours after eclosion, b) colonies may be so large that the resident male cannot fully control all brood piles, and c) queens produce so many males that not all are eliminated by the old male. LMC in single-queened nests of *C. obscurior* leads to the production of only very few new ergatoid males, while many more ergatoids are produced when multiple queens reproduce in the same nest [17,37]. We have previously argued that queens in multi-queen societies in this way may increase their chances of one of their own sons winning male-male competition [17]. The current study suggests that the presence of multiple pupae or freshly emerged ergatoid males might distract the resident male and thus increase the chance of individual queens that their own sons reach a well-fortified age undetected.

## Competing interests

The authors declare no competing interests.

## Authors’ contributions

SC and JH designed the study. SC and AS performed the behavioural observations and MS the gas chromatography analyses. SC analysed the behavioural data and JH the chemical data. SC, AS and JH wrote the paper. All authors read and approved the final manuscript.
